# Loss of Programmed cell death 4 (Pdcd4) associates with the progression of ovarian cancer

**DOI:** 10.1186/1476-4598-8-70

**Published:** 2009-09-03

**Authors:** Na Wei, Stephanie S Liu, Thomas HY Leung, Kar F Tam, Xiao Y Liao, Annie NY Cheung, Karen KL Chan, Hextan YS Ngan

**Affiliations:** 1 Department of Obstetrics & Gynaecology, Queen Mary Hospital, the University of Hong Kong, Hong Kong, PR China; 2 Department of Pathology, Queen Mary Hospital, the University of Hong Kong, Hong Kong, PR China

## Abstract

**Background:**

Programmed cell death 4 (Pdcd4) is a novel tumour suppressor and originally identified as a neoplastic transformation inhibitor. The aim of this study was to investigate the expression, prognostic significance and potential function of Pdcd4 in ovarian cancer.

**Results:**

The expression of Pdcd4 was examined in 30 normal ovarian tissues, 16 borderline and 93 malignant ovarian tissues. A continuous down regulation of Pdcd4 expression in the sequence of normal, borderline and malignant tissues was observed. The expressions of Pdcd4 in both ovarian borderline tissues and carcinomas were significantly lower than the expression in normal ovarian tissues (p < 0.001). Furthermore, patients with lower Pdcd4 expressions were found to have shorter disease-free survival (p = 0.037). The expression of Pdcd4 was also assessed by immunohistochemical analysis in 13 ovarian normal tissues and 44 carcinomas. Different subcellular localization of Pdcd4 was observed in normal compared to malignant cells. Predominant nuclear localization of Pdcd4 was found in normal ovarian tissues while ovarian carcinomas showed mainly cytoplasmic localization of Pdcd4.

**Conclusion:**

Our study demonstrated that the loss of Pdcd4 was a common abnormality at molecular level in ovarian cancer and it might be a potential prognostic factor in ovarian cancer patients.

## Background

Ovarian cancer is the 5^th ^commonest cause of cancer deaths in women in western countries according to the U.S. cancer statistics [1]. Ovarian cancer patients are often diagnosed at a late stage, which partially contributes to its poor prognosis. The mainstay of treatment includes cytoreductive surgery followed by adjuvant chemotherapy. Despite optimal primary treatment, recurrences are common and the overall prognosis is poor. The development of novel ovarian cancer diagnostic tests as well as treatment is urgently required.

Programmed cell death 4 (Pdcd4), a newly identified tumour suppressor, has been demonstrated to inhibit tumour promoter-induced neoplastic transformation in the murine JB6 cell model system [2]. It inhibited AP-1-dependent transcriptional activity [3], and directly interacted with and inhibited the helicase activity of eukaryotic translation initiation factor 4A (eIF4A) by competing its binding to the scaffold protein eIF4G, thus subsequently inhibited translation [4]. The human Pdcd4 gene was localized in chromosome 10q24 [5]. It was expressed in small duct epithelial cells of the normal mammary gland [5,6], normal human lung tissue [7], and senescent human fibroblasts [8]. It was commonly lost in lung cancer and such a loss was correlated with higher histological grade, disease stage and poor prognosis[7]. Expression of Pdcd4 was found to be reduced in hepatocellular carcinoma (HCC) [9].

Some studies investigating cellular functions of Pdcd4 demonstrated that it could regulate molecules such as p21 [10], Cdk4, ornithine decarboxylase [11], carbonic anhydrase II [12], and JNK/c-Jun/AP-1[13]. It has also been shown to suppress the expression of the invasion-related urokinase-receptor-(u-PAR)-gene, and to suppress invasion/intravasation via Sp1/Sp3 promoter motifs in cancer [14]. The ability of Pdcd4 to suppress colon carcinoma cell invasion may involve the downregulation of MAP4K1 transcription [15]. Stable expression of antisense Pdcd4 significantly reduced the sensitivity of MCF-7 breast cancer cells to geldanamycin and to tamoxifen [16].

Despite the above findings, the roles of Pdcd4 are not universal. The physiological roles of Pdcd4 in human cancers are still poorly understood and its role in ovarian cancer has not been thoroughly investigated. In this study, we aimed to quantify Pdcd4 mRNA and protein expressions in human ovarian normal, borderline and malignant tissues and to correlate the expressions with clinical and pathological parameters, including histology, grade, stage, disease free and disease specific survival, overall survival, as well as sensitivity to chemotherapy.

## Results

### Differential expression patterns of Pdcd4 in normal and malignant ovarian cells

**Figure 1:**
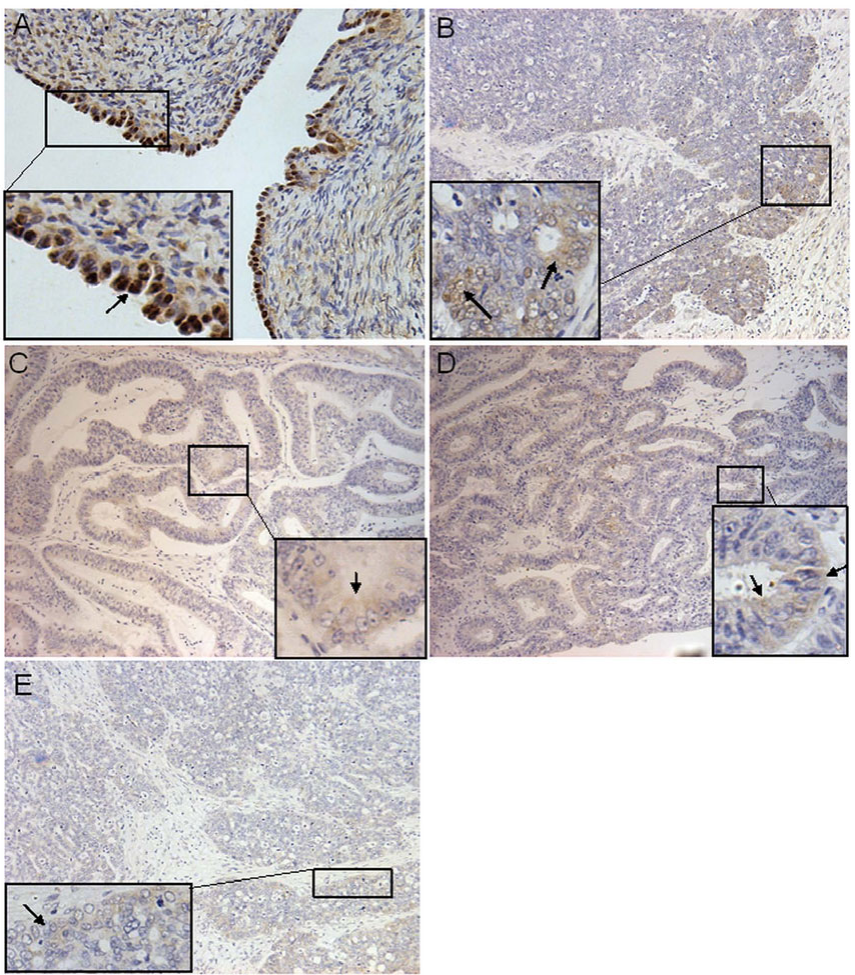
**Representative examples of immunohistochemical staining of Pdcd4 in ovarian tissues**. (A) Distinct nuclear staining was found in normal ovarian surface epithelium while much weaker cytoplasmic staining was found in (B) serous (C) mucinous, (D) endometrioid and (E) poorly differentiated adenocarcinomas. Arrows indicated representative positive staining sites.

Thirteen normal ovarian and 44 ovarian carcinoma paraffin tissue samples were available for immunohistochemical study. Among these samples, two out of the 13 normal and 26 out of the 44 malignant tissue samples were a subset of our sample pool for the western blot analysis. Positive Pdcd4 staining at the ovarian surface epithelium was observed in 11 out of 13 (84.6%) normal samples and 18 out of 44 (40.9%) carcinomas. Pdcd4 expression in all normal ovarian epithelial tissues (n = 11) showing positive staining was localized in nucleus (Figure 1A). On the other hand, among the cancer tissue samples showing positive staining, 6 out of 18 (33.3%) displayed nuclear localization, 9 out of 18 (50%) displayed cytoplasmic localization (Figure 1B C D E), and 3 out of 18 (16.7%) displayed both nuclear and cytoplasmic localization. There was a significant difference in Pdcd4 cellular localization between normal and cancer tissues (p = 0.002).

### Differential Pdcd4 expression levels between normal and malignant ovarian tissue samples

**Figure 2:**
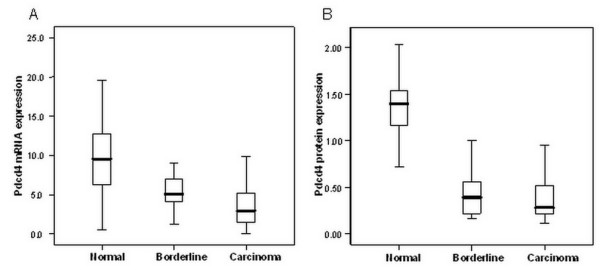
**Differential Pdcd4 mRNA and protein expressions in normal, borderline and malignant ovarian tissue samples**. (A) Pdcd4 mRNA expressions were significantly lower in malignant (median 3.07, interquartile range 1.36-5.83) when compared with borderline tissues (5.04, 3.95-7.18) (p = 0.041), and both malignant and borderline tissues showed significant lower expressions than normal tissues (9.54, 6.25-12.78) (p < 0.001 and p = 0.001 respectively). (B) Pdcd4 protein expressions were significantly lower in both borderline (median 0.39, interquartile range 0.21-0.57) and malignant (0.28, 0.21-0.49) ovarian cancer tissues when compared with normal tissues (1.39, 1.15-1.53) (p < 0.001).

Pdcd4 mRNA expressions were assessed in 30 normal, 16 borderline and 93 malignant ovarian samples by real time quantitative PCR analysis. There was a falling trend in Pdcd4 mRNA expression in the sequence of normal-borderline-malignant tissues. Pdcd4 mRNA expressions were significantly lower in malignant tissues (median 3.07, interquartile range 1.36-5.83) compared to borderline tissues (5.04, 3.95-7.18) (p = 0.041). Pdcd4 mRNA expressions in both malignant and borderline tissues were significantly lower compared to normal tissues (9.54, 6.25-12.78) (p < 0.001 and p = 0.001 respectively) (Figure 2A).

**Figure 3:**
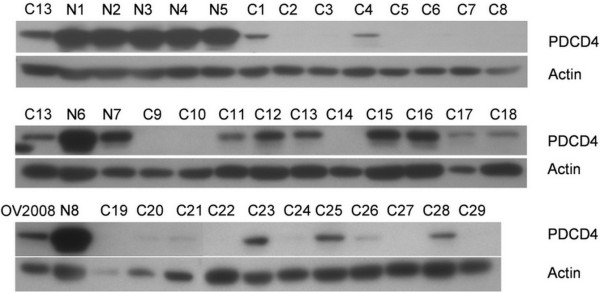
**Western blot analysis of Pdcd4 expression in normal ovarian and malignant ovarian tissue samples**. A representative of 8 normal samples (N1-8) and 29 malignant samples (C1-29) indicated significant higher Pdcd4 protein expression in normal compared with malignant ovarian tissue samples. Ovarian cancer cell line C13 or OV2008 were included as positive control and β-actin as loading control.

Pdcd4 protein expressions were assessed by western blot analysis. Pdcd4 was identified as a main band at around 60 kD (Figure 3). A similar falling trend in the sequence of normal-borderline-malignant tissues was also found in Pdcd4 protein expression. The expressions in both borderline (median 0.39, interquartile range 0.21-0.57) and malignant (0.28, 0.21-0.49) tissues were significantly lower compared to the expressions in normal ovarian tissues (1.39, 1.15-1.53) (p < 0.001) (Figure 2B). Pdcd4 mRNA expressions correlated with protein expressions according to western blot analysis (Spearman's rho = 0.515, p < 0.001).

IHC analysis also demonstrated similar results. Among the eleven normal ovarian samples with positive Pdcd4 expression, five (45.5%) showed strong expression; five (45.5%) showed moderate expression; one (9.0%) showed weak expression. On the other hand, among the nine carcinomas showing exclusive cytoplasmic staining, four (44.4%) showed moderate expression and five (55.6%) showed weak expression. Among the six carcinomas showing exclusive nuclear staining, one (16.7%) showed strong expression, two (33.3%) showed moderate expression and the rest (50%) showed weak expression. All three carcinomas with both cytoplasmic and nuclear staining showed weak expression.

Only 28 samples, including two normal and 26 malignant tissues, had both frozen and paraffin tissues available for western blot analysis and immunohistochemical staining. 20 out of 28 (71.4%) samples showed concordant results in both tests (pearson's correlation = 0.455, p = 0.015). Discordant results were observed in eight samples. Seven with positive western result showed negative IHC staining and one sample was vice versa.

### Correlation of Pdcd4 expressions with clinical parameters

**Figure 4:**
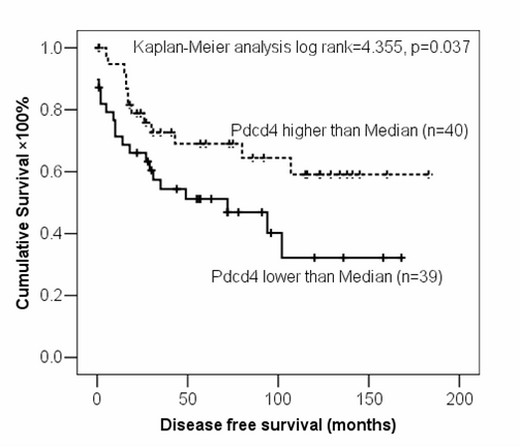
**Influence of Pdcd4 expression on disease-free survival of ovarian cancer patients**. Pdcd4 expression was categorized into lower or higher groups by median value. Pdcd4 was significantly associated with disease-free survival of ovarian cancer patients (n = 79, p = 0.037).

IHC staining correlated with western blot results, therefore, western blot results for Pdcd4 expressions were used in clinical correlation. For borderline tissues and carcinomas (n = 109), clinical data were collected from patients' records. The median follow-up period was 49 months (inter quartile range 26-97 months). The disease stages (stages I-IV) were significantly correlated with disease-free and overall survival (p < 0.001). Among ovarian cancer patients with known recurrence status (n = 79), by categorizing Pdcd4 expressions into two levels at median value (ie, lower expression <= 0.28, higher expression > 0.28), Pdcd4 was found to be significantly associated with disease-free survival (log rank = 4.355, p = 0.037) (Figure 4). Higher Pdcd4 expressions were associated with better disease free survival of ovarian cancer patients. In multivariable analysis using Cox proportional hazards regression analysis with age, stage of disease, grade and Pdcd4 expression as covariates, Pdcd4 remained as a significant predictor for disease-free survival (p = 0.007). Apart from Pdcd4 expression, stage of disease and age were also significant independent predictors for disease-free survival (p = 0.010 and p = 0.027, respectively) (Table 1). Using the same categorizing criteria, no correlation could be found between Pdcd4 expression and other clinical parameters including age, stages of disease, histological types, grade, metastasis, disease specific and overall survival (data not shown).

**Table 1:** Cox regression analysis for factors affecting disease free survival

**79 ovarian cancer patients (median as cut-off value)**		
**Variables**	**Relative risk (95% CI)**	**P value**
Age	2.583 (1.114-5.989)	**0.027**
Grade	1.616 (0.524-2.572)	0.713
Stage of disease	3.487 (1.347-9.022)	**0.010**
Pdcd4	0.292 (0.120-0.711)	**0.007**

CI: confidence interval; Coding of variables: Age was coded as 0 (Age <= 50) and 1 (Age > 50);

FIGO stage was coded as 0 (stage I and II) and 1(stage III and IV); Grade was coded as 0 (grade I and II) and 1(Grade III); Pdcd4 expression was coded as 0 (Pdcd4 expression <= 0.28) and 1 (Pdcd4 expression > 0.28).

P value in bold indicated statistical significance.

Among 71 ovarian cancer patients treated with platinum chemotherapy, 68 of them had platinum therapy after surgery. 55 out of 71 (77.5%) were platinum sensitive and the others were platinum resistant. 31 of 55 (56.4%) platinum sensitive patients had higher Pdcd4 expression (above median value) and 6 of 16 (37.5%) platinum resistant patients had higher Pdcd4 expression. There was no significant difference in the proportion of patients with higher Pdcd4 expression between the platinum sensitive and resistant groups.

## Discussion

In the present study, Pdcd4 mRNA and protein expressions were assessed in a total of 30 normal, 16 borderline and 93 malignant ovarian tissues. A decreasing trend of both mRNA and protein expression of Pdcd4 in the sequence of normal-borderline-malignant ovarian tissue samples was demonstrated and the expressions of Pdcd4 were significantly reduced in both borderline and malignant samples. This suggested that Pdcd4 might play a role in human ovarian carcinogenesis. Our results were consistent with the findings in human hepatocellular carcinoma and lung cancer where loss of Pdcd4 expression was found in cancer compared with normal tissues [7,9]. However, a study from Yoshinaga et al showed that increased Pdcd4 expression was detected in bladder cancer, suggesting that cell type or tissue specific function of Pdcd4 may exist [6]. Our data demonstrated a good correlation between Pdcd4 mRNA and protein expression in ovarian tissue samples (p < 0.001), which was different from the findings in lung tumours by Kalinichenko and colleagues [17]. This may be either due to a tissue specific property of Pdcd4 or the different quantification approaches between Kalinichenko's and our study, in which we used real time quantitative PCR instead of conventional semi-quantitative PCR.

Loss of Pdcd4 expression had been demonstrated to correlate with tumour grade, disease stage, recurrence and patient survival in lung and colorectal cancers [7,18]. In our study, by classifying Pdcd4 expression into two groups using median as a cut-off value, we found that patients with higher Pdcd4 expression had better prognosis in terms of disease-free survival, which implied that Pdcd4 might be a potential prognostic factor to predict the outcomes of ovarian cancer patients. However, we did not find any significant correlation between Pdcd4 expression and overall survival. Since the majority of the death cases (40 of 43) in our series were tumour related, there was no significant correlation between Pdcd4 expression and the disease specific survival either. High Pdcd4 level might prolong the time to recurrence after initial treatment. However, once the disease recurred, the protective effect of Pdcd4 might be limited and other molecular events could have overtaken, and the tumour somehow becomes more resistant to the treatment, such as second line chemotherapy. This might lead to the non-significant difference in the overall survival in the two groups. Such speculations would need to be confirmed and the mechanisms involved would need to be elucidated in future studies.

A study in breast cancer cell line demonstrated knockdown of Pdcd4 significantly reduced the sensitivity to geldanamycin and tamoxifen [16]. Therefore, we also tested whether there was an association between Pdcd4 expression and platinum sensitivity of ovarian cancer. We chose platinum for our correlation because it was the most commonly used first line chemotherapy for ovarian cancer patients. According to our results, no correlation of Pdcd4 expression and platinum sensitivity could be found.

When this manuscript was being prepared, another study on Pdcd4 in ovarian cancer by Wang et al was published. There were a number of differences between their study and ours. They applied semi-quantitative PCR to measure Pdcd4 mRNA expression in serous subtype of ovarian cancer tissues. Instead, we applied real time PCR which enabled us to detect Pdcd4 mRNA expression more precisely and we have included various subtypes such as serous, mucinous, endometrioid, clear cell, etc. They demonstrated that the loss of Pdcd4 expression was associated with the progression of serous cystadenocarcinoma [19]. We applied subgroup survival analyses on patients with serous and endometrioid subtypes. However, no significant correlation of Pdcd4 expression with disease free survival was found (data not shown) when using the median expression as the cut-off value. The insignificant results might be due to the smaller sample size in each subgroup. The number of samples for the other histological types such as clear cell, mucinous, and mixed type was too small for individual statistical analysis. Our results demonstrated that the localization of Pdcd4 in normal tissues were distinctively in nucleus, which again was different from their findings.

Several studies have been conducted to investigate the role of Pdcd4 during tumour progression. Overexpression of Pdcd4 resulted in inhibition of carcinoid cell proliferation [20]. The anti-proliferative effect of Pdcd4 was demonstrated through the repression of transcription of the mitosis-promoting factor cyclin-dependent kinase (CDK)1/cdc2 via upregulation of p21^Waf1/Cip1 ^in human carcinoid cells [10]. Pdcd4 transgenic mice showed lower tumour incidence and papilloma-to-carcinoma conversion frequency compared with wild-type mice [11]. Pdcd4 was also suggested to be a proapoptotic molecule involved in TGF beta-1 induced apoptosis in HCC cells [9]. Diminished Pdcd4 expression deregulated the normal DNA-damage response thus preventing DNA-damaged cells from undergoing apoptosis [21]. Using a mouse skin carcinogenesis model, Schmid et al demonstrated that Pdcd4 limited tumour formation in vivo, and it was targeted for degradation during tumour promotion [22]. Dorrello's study demonstrated that degradation of Pdcd4 in mitogen-stimulated cells was required for efficient protein translation thus essential for cell growth or proliferation [23]. Phosphorylation of Pdcd4 by AKT was essential for induction of ubiquitin-mediated Pdcd4 degradation [24]. As it has been suggested that the role of Pdcd4 might be cell type specific [25], the regulatory mechanisms of Pdcd4 in ovarian cancer cells still remains to be investigated.

The shuttle of Pdcd4 between nucleus and cytoplasm has been proposed to have important effect on regulation of its function. Pdcd4 localized predominantly in nucleus under normal growth conditions but was exported to the cytoplasm upon serum withdrawal [26]. A number of groups have reported the localization of Pdcd4 in different cell types. Yoshinaga et al found that Pdcd4 accumulated in the nucleus at the G0 phase of asynchronous cultures of human normal fibroblasts but was localized in the cytoplasm during the cell cycle in tumour cell lines [6]. According to Goke's study, epithelial cells of prostate, breast and lung disclosed intense nuclear but no cytoplasmic staining; a clear shift from nuclear localization to cytoplasmic staining was observed in all colonic adenomas investigated. In the case of invasive breast cancer tissues, both nuclear and cytoplasmic localization were observed [10]. As proposed by Zhang [9], the accumulation of Pdcd4 in the nuclei was important for apoptosis, and the regulatory mechanisms of the localization of Pdcd4 protein may play an important role in the induction of apoptosis in HCC cells. In an investigation conducted in colorectal cancer, besides decreased expression level of Pdcd4, a significant loss of nuclear Pdcd4 from normal tissues to colonic adenomas and carcinomas was also observed [18], which again supported the notion that the intracellular localization of Pdcd4 might play an important regulatory role in tumour cell progression. In this study, we also demonstrated similar results with other groups that a differential expression pattern of Pdcd4 was found between normal and carcinoma cells by IHC analysis. Normal ovarian tissue showed distinctive nuclear staining, whereas majority of cancer tissues displayed cytoplasmic staining. Our results suggested that Pdcd4 might translocate from the nucleus to the cytoplasm during ovarian cancer development. We speculated that the accumulation of Pdcd4 in the nucleus might negatively regulate cell proliferation while the cytoplasmic sequestration of Pdcd4 might abolish its function in ovarian cancer cells. However, this phenomenon needs to be further confirmed in a larger sample pool, and in-vitro studies would be needed in order to understand its regulator mechanism. In conclusion, both localization and expression level of Pdcd4 might be the potential indicators of ovarian tumour progression.

## Conclusion

Our present study identified Pdcd4 as an ovarian cancer associated gene based on its differential expression patterns between normal and malignant ovarian samples. Reduction in Pdcd4 expression correlated with shortened disease-free survival suggesting that it might have a potential role in tumour suppressor. In addition, we hypothesized that Pdcd4 might translocate from the nucleus to the cytoplasm in malignant ovarian tissues. However, the regulatory mechanisms of Pdcd4 in ovarian tumour progression remained to be further investigated. Nonetheless, our study indicated that the loss of Pdcd4 was a common abnormality at molecular level in ovarian cancer and it could be a potential prognostic marker in ovarian cancer patients.

## Methods

### Sample collection

Ovarian tissues were obtained at the time of surgery between 1990 and 2006 and snap frozen in liquid nitrogen at the Department of Obstetrics and Gynaecology, Queen Mary Hospital, The University of Hong Kong. A total of 30 normal, 16 borderline and 93 malignant ovarian tissue samples were randomly selected. The histology of all the samples was confirmed and the tumour cell contents were at least 70%. The mean age (± standard deviation) of patients with normal ovaries, borderline and carcinomas were 56 years (± 11.4), 43 years (± 15), and 50 years (± 12), respectively. The histological types and disease stages were classified according to International Federation of Gynaecology and Obstetrics (FIGO) classification. The background characteristics for all the patients were summarized in Table 2. All 30 normal ovarian samples were obtained from women who had undergone hysterectomy for benign diseases.

**Table 2:** Background characteristics for all recruited ovarian cancer patients

**Characteristic**	**Borderline (n = 16)**	**Carcinoma (n = 93)**
Age	43 (± 15)	50 (± 12)
stage		
1	10 (62.5)	30 (32.2)
2	1 (6.3)	15 (16.1)
3	2 (1.3)	37 (39.8)
4	0	9 (9.7)
unstaged	3 (18.8)	1 (1.1)
unknown stage		1 (1.1)
Histology		
Endometrioid		25 (26.9)
Mucinous	13 (81.3)	10 (10.7)
Serous papillary	3(18.7)	29 (31.2)
Clear cell		14 (15.0)
Mixed		12 (12.9)
Undifferentiated		3 (3.2)
Number of recurrences	5 (31.2)	35 (37.6)
Number of persistent disease	0	14 (15.0)
Disease-free interval (months)	49 (30-110)	28 (6-79)
Overall survival time (months)	56 (40-110)	46 (24-97)
With platinum treatment	1 (6.3)	70 (75.3)
Sensitive to platinum	1 (100)	54 (77.1)
Resistant to platinum		16 (22.9)
Without platinum treatment	15 (93.7)	23 (9.1)

The use of clinical specimens was approved by the local institutional ethics committee (institutional review board reference No: UW 05-143 T/806). Patients with ovarian cancer were treated according to our unit's protocol which consisted of total abdominal hysterectomy, bilateralsalpinpoophorectomy (BSO) and staging procedure for early disease, or debulking for advanced disease followed by adjuvant chemotherapy which consisted of either carboplatin alone or in combination with paclitaxel. Disease free interval was defined as the time from surgery/end of chemotherapy to either disease progression, recurrence, death or the last follow-up date of the patients. Response to platinum therapy was defined as platinum sensitive or resistant when the recurrence occurred after or within six months following the completion of therapy respectively. Patients were considered as platinum refractory if they did not achieve complete response after the platinum therapy, and they were also considered as platinum-resistant.

### RNA extraction and real time quantitative PCR

Total RNA was extracted from the collected tissues using TRIZOL^® ^Reagent (Invitrogen corporation, Carlsbad, CA). cDNA was then synthesized from 1 μg of total RNA using Superscript III reverse transcriptase (Invitrogen). Pdcd4 mRNA expression was quantitated by real time quantitative RT-PCR (ABI Prism 7500) using power SYBR^® ^Green PCR Master Mix (Applied Biosystems, Foster, CA) and specific primers for Pdcd4 (forward: ATGATGTGGAGGAGGTGGATGTG; reverse: CCAATGCTAAGGATACTGCCAAC). An endogenous control, TATA box Binding Protein (TBP), was used to normalize Pdcd4 expressions. The standard curve was constructed from C_T _values using 5 serial 10-fold dilutions of linearized plasmids of Pdcd4 and TBP, respectively, and the absolute copy numbers of Pdcd4 mRNA was then determined. All measurements were conducted in duplicates. Results for the quantity of mRNA were expressed as the number of copies of Pdcd4 mRNA per copy of TBP mRNA.

### Protein extraction and western blot analysis

Tissue sample proteins were extracted using the conventional method. Briefly, protein extraction RIPA buffer (NaCl 0.15 M, Tris-HCl 0.05 M, NP-40 1%, DOC 0.005 g/ml, SDS 0.1%) supplemented with proteinase inhibitor PMSF 1 mM, Aprotinin 1 ug/ml and Leupeptin 2.5 ug/ml (Sigma, St Luis, MO) were added to the homogenized tissue samples. Protein lysates were collected after centrifugation and subjected to the subsequent western blot analysis. Extracts containing 20 ug protein were electrophoresed in 10% polyacrylamide-SDS gels and transferred onto PVDF membranes (Bio-Rad Laboratories, Hercules, CA). The membrane was blocked with 5% nonfat milk in TBST (Tris-HCl 5 mM, pH 7.5, NaCl 0.15 M, Tween20 0.1%) at room temperature for one hour and incubated with affinity purified Pdcd4 antibody (Rockland, Gilbertsville, PA) at a dilution of 1:2500 overnight at 4°C. After intensive wash with TBST, membrane was then incubated with horseradish peroxidase (HRP) conjugated donkey anti-rabbit secondary antibodies (Amersham Biosciences, Buckinghamshire, UK) for one hour at room temperature. Beta-actin was routinely detected as the internal control using the beta-actin primary antibody (Sigma) at a 1:5000 dilution and horseradish peroxidase (HRP) conjugated sheep anti-mouse secondary antibodies (Amersham). The membrane was then developed by ECL plus (Amersham). The relative amount of Pdcd4 protein expression was quantitated by densitometric scanning and normalized by actin intensity.

### Immunohistochemistry

Formalin-fixed and paraffin-embedded clinical samples were retrieved from the Department of Pathology, Queen Mary Hospital, The University of Hong Kong. They were sectioned at 5 μm thick and mounted on aminopropyltriethoxysilane-coated (Sigma, Saint Louis, MO) slides. Histopathological types were evaluated by pathologists and the consecutive section was subjected to immunohistochemical staining. Briefly, the slides were deparaffinized, re-hydrated, and then immersed in distilled water with 3% hydrogen peroxide to suppress endogenous peroxidase activity. Antigen retrieval was performed by microwave treatment in 0.01 M citrate buffer (pH 6.0) for 15 minutes. Sections were incubated with Pdcd4 antibody (Rockland) at a dilution of 1:100 in 5% normal goat serum (Zymed Laboratories Inc, South San Francisco, CA) overnight at 4°C. Envision peroxidase-labeled polymer (goat-anti-rabbit) (Dako North America Inc, Carpinteria, CA) was then applied to the sections and signals were developed by chromogen diaminobenzidine (Amresco Inc., Salon, OH). The immunoreactivity was estimated and graded by scoring the percentage of positive nuclear or cytoplasmic staining (negative, <=5%; weak, 5-19%; moderate, 20-49%; strong, 50-100%) and intensity of cytoplasmic staining (negative, weak, moderate, strong).

### Statistical analysis

Box plots were used to describe Pdcd4 expression levels in normal, borderline and malignant ovarian tissue samples where the box stretched from 25^th ^percentile to 75^th ^percentile. A line cross the box indicated the median value, while error margins indicated the smallest and largest outliers. Non parametric Kruskal-Wallis test was used to compare Pdcd4 expression differences among normal, borderline and carcinomas groups; Mann-Whitney U test and Wilcoxon signed rank test were used to compare differences between two groups. Crosstabs and Pearson Chi-Square were used to test the correlation between Pdcd4 protein expression and clinical parameters. Non parametric Spearman's rho's correlation was used to test the correlation of Pdcd4 mRNA and protein expression. Disease free survival analysis was carried out by Kaplan-Meier and compared by log rank test. Multivariable analysis (Cox's regression model) was used to adjust for confounding factors that might affect the outcome. All statistical analysis was performed by Statistical Package for Social Science (SPSS, v13, SPSS Inc., Chicago, IL) and significance was assumed if p < 0.05.

## Abbreviations

Pdcd4: Programmed cell death 4

## Competing interests

The authors declare that they have no competing interests.

## Authors' contributions

NW performed RNA and protein extractions, Real time quantitative PCR, western blotting, Immunohistochemical staining, statistical analysis and prepared the manuscript. SSL coordinated the study, interpreted the results and revised the manuscript. KKLC and KFT collected the clinical data. XYL performed IHC scoring. THYL contributed to the scientific discussion and the manuscript editing. ANYC provided the paraffin tissue blocks and supervised IHC data analysis. HYSN, SSL, and KKLC supervised in the design of the study and finalized the manuscript. All authors read and approved the final manuscript.
